# Aspects of well-being when struggling with obesity

**DOI:** 10.1080/17482631.2019.1699637

**Published:** 2019-12-06

**Authors:** Britt Marit Haga, Bodil Furnes, Elin Dysvik, Venke Ueland

**Affiliations:** Faculty of Health Sciences, University of Stavanger, Stavanger, Norway

**Keywords:** Obesity, phenomenology, well-being, health, lifeworld, lived experiences, existential experiences

## Abstract

**Purpose:** We aimed to gain deeper insight into how people struggling with obesity handle their life situation by addressing how well-being might unfold. For many people, obesity becomes a lifelong condition characterized by repeated weight fluctuations while their weight increases gradually. From an existential perspective, constantly waiting for weight loss can cause an experience of not reaching one’s full potential. How people with obesity experience well-being, within their perceived limitations, is less reflected in previous research.

**Methods:** We established a qualitative study using in-depth interviews with seven men and 14 women with obesity (body mass index ^3^35 kg/m^2^) aged 18–59 years. The study had an exploratory design including a phenomenological–hermeneutic perspective, with a lifeworld approach.

**Results:** Three themes describing aspects of well-being were developed: *coming to terms with the body, restoring the broken relational balance and reorienting the pivot in life*. The thematic findings were abstracted into a main theme: striving to make living bearable. The movement towards well-being can be seen as a struggle towards an experience of balance to make bearable living.

**Conclusions:** We suggest that well-being as a dialectic between vulnerability and freedom might become a health-facilitating experience for people struggling with obesity.

## Introduction

The worldwide prevalence of people with obesity has nearly doubled since 1980 (World Health Organization [WHO], 2014), and many of those affected make repeated attempts to lose weight (Bombak & Monaghan, 2017; Owen-Smith, Donovan, & Coast, 2014). Losing weight and maintaining weight loss over the long term seems to be difficult (Look AHEAD Research Group, 2014). Hence, obesity becomes a lifelong condition often characterized by repeated weight fluctuations while the weight gradually increases (Grønning, 2014). In the light of modern trends for individualism and striving for self-fulfilment, this life situation while waiting for weight loss,—trying and/or wanting to lose weight,—can cause feelings about not reaching one’s full potential and desires in life (Glenn, 2013; Grønning, 2014; Ueland, 2019). People struggling with obesity cannot escape their cultural context, or experienced limitations; they can only find a way of handling them (Malterud & Ulriksen, 2010; Ueland, 2019).

How people challenged with illnesses such as obesity handle life within their experienced limitations is considered to have strong influence on the individual’s perceived well-being, and on their potential to move towards health (Dahlberg, Dahlberg, & Nyström, 2008). Health can be found in illnesses as a balance between suffering and well-being (Todres, Galvin, & Dahlberg, 2014). However, well-being as the individual’s felt experience of feeling well, that is living the life they desire, and being able to carry out minor and major life projects (Dahlberg & Segesten, 2010), is reflected to a lesser extent by the expanding field of obesity studies.

The current dominant approaches to obesity involve lifestyle changes (diet and exercise modifications) cognitive behavioural therapy, weight loss medication and bariatric surgery (Bray, Frühbeck, Ryan, & Wilding, 2016). However, even after having sought treatment, research has shown that people living with obesity continue to struggle with existential issues (Merrill & Grassley, 2008; Rørtveit, Furnes, Dysvik, & Ueland, 2017). Therefore, these approaches do not seem to capture the existential struggle of living and handling life with obesity. As the long-term maintenance of weight loss is so limited and uncertain, and the implications for physical health are questionable, studies are increasingly addressing other possible approaches to obesity (Logel, Stinson, & Brochu, 2015; Look AHEAD Research Group, 2014; Samdal & Meland, 2018). Some studies suggest that obesity management and policies should better address mental well-being, and even prioritize well-being over weight loss (Rand, Vallis, Aston, Price, Piccinini-Vallis, Rehman & Kirk 2017; Tylka, Annunziato, Burgard, Danielsdóttir, Shuman, Davis & Calogero 2014). People with obesity themselves seem to highlight their health outcome as being more important than simple appearance (Sand, Emaus, & Lian, 2015).

Studies have shown that when dedicated to health and well-being, people with obesity develop multifaceted coping mechanisms for dealing with stigma and misunderstanding from society (Bombak, 2015; Puhl & Brownell, 2003). Hence, a holistic approach is considered to optimize health and well-being among people with obesity by turning attention towards their own resources to strengthen empowerment and well-being (Borge, Christiansen, & Fagermoen, 2012; Brown & Wimpenny, 2011; Knutsen, 2012: Kristjansdottir, Stenberg, Mirkovic, Krogseth, Ljoså, Stange & Ruland, 2018).

The importance of well-being outcomes in addition to weight loss in connection with obesity treatment has been underlined in several studies (Palmeira, Pinto-Gouveia, & Cunha, 2017; Willmott & Parkinson, 2017). Positive results from transdisciplinary interventions based on non-dietary principles, focusing on respecting one’s body shape, size diversity and promoting a holistic approach towards wellness, might guide future approaches (Clifford, Ozier, Bundros, Moore, Kreiser & Morris, 2015; Samdal & Meland, 2018).

Thus, as a parallel to the obesity field, it seems relevant to compare it to the scientific approach to living with chronic illnesses in general. How people with chronic illnesses handle life and experience well-being despite their suffering has been examined widely (Delmar, Boje, Dylmer, Forup, Jakobsen, Moller & Pedersen, 2005; Johansson, Österberg, Leksell, & Berglund, 2015; Kristjansdottir et al., 2018). The dominant perspective is how people with chronic illnesses find meaningful ways to live with their challenges, despite their perceived limitations. Those people who manage to preserve or create meaning in life, in spite of pain or disabilities, are also able to reformulate their lifeworld and live a full life (Sjöling, Ågren, Olofsson, Hellzén, & Asplund, 2005).

Some recent studies have enhanced the understanding of what it is like to live with obesity as an existential challenge (Haga, Furnes, Dysvik, & Ueland, 2019; Ueland, Furnes, Dysvik, & Rørtveit, 2019; Westland Barber, 2017); however, these studies did not examine how people with obesity live their life in spite of their challenges. As far as we have found, very few studies have provided an existential perspective, describing how people with obesity might experience well-being within their perceived limitations. However, some phenomenological studies highlighting efforts to maintain weight loss are relevant to our study. These have reflected upon how people strive for balance, identity and meaning in life, within their challenges of maintaining long-term weight loss (Groven, Galdas, & Solbrække, 2015; Groven, Råheim, & Natvik, 2017; Natvik, Gjengedal, Moltu, & Råheim, 2015; Toft & Uhrenfeldt, 2015; Warholm, Øien, & Råheim, 2014). We see this striving as a parallel to people’s struggle to handle a challenging life when living with obesity. Toft and Uhrenfeldt (2015) suggested addressing the experience of well-being when intervening to deal with obesity.

Todres and Galvin (2010) emphasized that well-being is both a way of being in the world as well as a felt sense. They described the deepest existential well-being as “dwelling-mobility”, suggesting that our existence in the world needs both a foundation and a movement forward. Drawing on a conceptual framework, Galvin and Todres (2011) described 18 kinds of well-being experiences in which dwelling and mobility occur with different emphases. The lifeworld constituents elaborated by Heidegger contain the descriptions of spatiality, temporality, intersubjectivity, mood and embodiment (Heidegger, 1962). These 18 kinds of well-being experiences can lead to the formulation of resources that have the potential to give rise to well-being as a felt experience, and thus guide new directions for caring. Hence, an existential theory of well-being is about its structure before it is divided, e.g., into the dimensions of physical, emotional or social well-being (Hörberg, Ozolins, & Ekebergh, 2011; Todres & Galvin, 2010).

Based on the above considerations, we need to develop more knowledge about how people handle their life when struggling with obesity and which aspects of well-being might manifest in such situations. Exploring their ways of handling life can reveal how the person with obesity make a bearable living, within their limitations and vulnerability. Hence, the aim of this study was to gain deeper insight into how people living with obesity handle their life situation by addressing the key question: in what way does well-being unfold within the struggle of living with obesity?

## Methods

This qualitative study had an exploratory design including a phenomenological/hermeneutic perspective (Kvale & Brinkmann, 2009). Our approach was open to the lived experiences of people with obesity and was therefore based on a *lifeworld* approach. Lifeworld is defined as the world of lived experiences in which we live our lives (Dahlberg et al., 2008). The lived experiences should be examined as described by the subjects and represents the phenomenological idea of going “to the things themselves” (Dahlberg et al., 2008, p. 32).

### Recruitment and participants

A convenient sample of 21 people (seven men and 14 women) was included in the study, representing those to whom we had access, with a willingness to share their life experiences according to the phenomena of interest (Malterud, 2011), which in our study was well-being within the struggle of living with obesity. The participants were recruited shortly after their entry into one of two different health promotion programmes, promoting lifestyle changes. We emphasized that our study dealt with experiences with life itself when struggling with obesity, and was not aimed at evaluating the programme. The programmes addressed different groups regarding medical severity and geographical catchment areas. The participants needed to meet the following recruitment criteria: age >18 years, both genders, ability to communicate in Norwegian, both orally and in writing, having a body mass index (BMI) ≥35 kg/m^2^, and able to provide informed consent on their own behalf. Table I presents the sample characteristics.

**Table I.** Sample characteristics (*N* = 21).

Sex	
Male	7
Female	14
Age, years	
18–29	6
30–39	2
40–49	3
50–59	10
Marital status	
In a relationship	11
Single	10
Education (highest level)	
Primary school	1
Secondary school	12
University/college	8
Employment	
Active	10
Temporary unemployment	10
Lasting unemployment	1
Weight	96–155 kg
BMI	35–51
Health-related suffering because of obesity	15
Obesity-reducing actions	
On their own	19
Healthy life centre (municipal level)	10
Intensive lifestyle intervention (specialist level)	16
Bariatric surgery	1
Weight-reducing medication	2
Other (group therapy, psychological intervention, etc.)	1

Before the inclusion, the leading health professionals in the programmes informed the candidates about the study, both orally and in writing, and a written consent form was completed. The first author contacted those who consented to the study and an appointment for the interview was made. Ethical approval was obtained from the Regional Committees for Medical and Health Research ethics (reference number 2016/1530), and the Norwegian Centre for Research Data (project number 50184) and conducted in accordance with the Helsinki Declaration (The World Medical Association, 2013).

It was emphasized that participation was voluntary and that the participants could leave the project at any time. Confidentiality was guaranteed, together with information about professional support for follow-up if needed.

### Data collection

We used qualitative interviews to deepen our insight into the aspects of well-being within the struggle of living with obesity, because this phenomenon is tied to human existence (Dahlberg et al., 2008; Kvale & Brinkmann, 2009). The interviews were conducted using a thematic interview guide with topics including: Everyday life with obesity and handling life with obesity, a meaningful life and future possibilities.

The participants were encouraged to deepen their reflections through follow-up questions, such as “Can you please tell me more … ?” and “What did you feel … .?” Each participant was given a choice to conduct the interview where they felt safe. Seventeen of the participants were interviewed in an office or a conference room at the health promotion centre; three interviews were conducted in the first author’s office and one in the first author’s private home. The interviews lasted 40–90 minutes. One person did not turn up for the interview. After having interviewed 21 participants, the research team concluded that the sample size probably had sufficient information power to elucidate the aim of the study, so the recruitment process was ended (Malterud, Siersma, & Guassora, 2016).

### Analysis and interpretation

The interviews were undertaken by the first author, audio-recorded and transcribed verbatim, 16 interviews by the first author and five by a professional transcriber who signed a confidentiality agreement in advance.

The data material was analysed inspired by the phenomenological-hermeneutic thinking of Ricoeur (Delmar et al., 2005; Furnes & Dysvik, 2012; Pedersen, 1999; Ricœur, 1976; Schultz, Qvist, Mogensen, & Pedersen, 2014). The research team independently read and re-read the transcripts of the interviews to become an initial overview after which they met to discuss the material as a whole. Based on the discussion the first author undertook the structural analysis. All authors contributed accordingly to the further analysis and interpretation. As Ricoeur claims, reading a text is the dialectic between two attitudes, explanation and understanding (Furnes & Dysvik, 2012; Ricœur, 1976). In-depth analysis of findings were made by moving back and forth between the empirical level and the analytical level, between understanding and explanation of the text (Ricœur, 1976).

The analysis and interpretation followed three steps:

1. A naïve reading. Reading and re-reading was carried out to gain an overall view of the interview texts, and to obtain a holistic understanding of the meaning content, i.e., “what is said?”
2. Structural analysis. A structural analysis clarifies the dialectic between understanding (what is said?) and the explanation (what is spoken about?) with the purpose of making a deeper critical interpretation possible. Interpretation of the structural analysis and an understanding of the content led to the development of three themes.
3. Critical interpretation and discussion. The naïve reading and the structural analysis guided the way to the selection of theory. Our critical interpretation aimed at developing new understanding.

A presentation of the themes is presented first, followed by further discussion and interpretation.

## Findings

The presentation of our findings illuminates three themes: *coming to terms with the body, restoring the broken relational balance*, and *reorienting the pivot in life*, each of them derived from the abstraction process of the two units of significance for each theme. Quotes are provided to give the participants a clear voice. Table II presents an example from the analysis process.

**Table II.** Example from the analysis process covering one main theme and three themes.

Striving to make living bearable					
Coming to terms with the body		Restoring the broken relational balance		Reorienting the pivot in life	
Units of meaning(What is said?)	Units of significance(What is being spoken about?)	Units of meaning(What is said?)	Units of significance(What is being spoken about?)	Units of meaning(What is said?)	Units of significance(What is being spoken about?)
I probably don’t choose activities that imply a lot of movement. I frequently go to cafés, that kind of thing. I would rather attend art exhibitions, that’s more *me*. I would rather that than a walk in the woods, because the physical activity becomes too challenging. (Woman A 58 years old)	Negotiating with the body’s possibilities	Maybe the people at work see that I struggle with some things that they manage easier. For example, walking down a stone staircase carrying cardboard (*packaging*) and that kind of thing. I always say “I will just put it (*the packaging*) here” when I am unpacking goods. I don’t take one box down the stairs at a time. The exercise is good for me, but I don’t do it because of my hurting knee and the ankles. They laugh then. Oh well. But then I tease them afterwards, «I see that you don’t do it either». So I get it. (Woman B 58 years old)	Disarming the experience of degradation	I will always be very big, but as long as my body can take me up some mountaintop … (*stops in the middle of the sentence, thinks for a moment before he goes on*), or at least down to the sea and along the beach, I will be very satisfied and happy. (Man 54 years old)	Rethinking how life can be lived

### Coming to terms with the body

The participants described their relationship with their large body in varying terms. They knew that they needed to care about their bodies, but at the same time, they had trouble acknowledging their body fully. Our main impression was that the participants seemed to experience their body and life to be somehow separate. However, the participants’ statements expressed that they were in a process of coming to terms with their body, by gaining new insight about their bodies and themselves.

#### Discovering their body as a prerequisite for life

The participants revealed that they were in a process of discovering their body as a prerequisite for life. At the same time as relating to their body as something they would refrain from, they realized that their body was essential if they were to live the life they wanted. A 54-year old man first described his body as a heavy appendage he had to carry around, which he considered as not necessary to live his life. He stated: “*I don’t really need my body in life …* ” However, during the interview he reflected further on, realizing that his body was a prerequisite for life and that he had to change his mind set and his attitude to his body. Instead of neglecting it, he should take care of it if he should have the chance to live the life he desired:

If I don’t have my body with me, I don’t have my brain either, because they are still connected, to put it like that. So, I must take care of it … You must realise that your body is your best friend, because that’s what gets you around so that you can do all those fun things.

The statements “they are still connected” and “that’s what gets you around so that you can do all those fun things” expressed how the participants realized that they had access to life itself through their bodies. The body, no matter how much it also felt like a limitation for life, was also considered as a sort of companion to carry out minor and major life projects. The participants realized therefore that they had to consider coming to terms with their body and to be aware of it if they wished to achieve a good life.

Another 54-year-old man used a comparison with team play to describe his discovery of his body as a prerequisite for life. He stated: “*Now I’m no longer fighting against my body; I’m fighting with it*.” This phrase typified the participants’ shifted experience of their bodies. Earlier they had related to their bodies as enemies they had to fight against, but now they had gained the realization that they were members on the same team as their bodies. The body was no longer considered as an opponent to a better living, but a teammate. Teaming up with the body seemed essential for participants to have a good life.

#### Negotiating with the body’s possibilities

The participant’s considerations about their body uncovered that they were in a way negotiating with the possibilities of their body when figuring out what their bodies were capable of despite their limitations. A 37-year-old woman with a physically challenging health provider job in a homecare facility for people with mental retardation first referred to the drawbacks of having a large body in her daily work: “*I have some very fit colleagues, and they manage to get things done as easy as anything. Things are perhaps a bit slower for me*.”

However, in the next moment she also outlined some benefits, because a strong and powerful body was very helpful when being challenged physically by the residents:

My colleague and I have found that there are advantages to being large in this job. They (those taken cared for) can’t move mountains (laughs)! There are colleagues who have said, when it comes to enduring certain situations, they would not have managed as well as us.

To experience that others recognized their bodies to be strong, seemed to encourage the participants to look at their bodies more positively. This might explain their willingness to experience meaning and coherence in life. They certainly experienced bodily drawbacks, but they were also able to recognize their large body for its benefits.

The participants had tried different leisure activities during life with varying degrees of success due to their large bodies. A feeling of defeat was a common experience. However, some participants explained that during these experiences they had understood more about not only their body’s limits and capabilities, but also themselves. During the interview, a 58-year-old woman came to the realization that leisure activities, which made her feel well were more in accordance with the perception she had of who she was as a person:

I probably don’t choose activities that imply a lot of movement. I frequently go to cafés, that kind of thing. I would rather attend art exhibitions, that’s more me. I would rather that than a walk in the woods, because the physical activity becomes too challenging.

Making a change in which activities one could participate in according to what the body could manage, did not mean only movement away from one’s desires. Movement also occurred towards something new and desirable that had the potential to be of great significance to the individual. These kinds of reflections seemed to indicate that negotiation with the body’s possibilities had ended, and been replaced with an experience of harmony.

### Restoring the broken relational balance

The participants seemed to have strategies to defend themselves from the perceived spoken or unspoken judgements from others, whether it came from relatives, friends, colleges or the general society. Strategies for defending themselves were highlighted several times during the interviews as a way to handle the underlying judgemental tone and hereby restore the broken relational balance between them and others. It was about an attitude towards how one should react to other’s condemnation.

#### Disarming the experience of degradation

One of the strategies to restore the broken relational balance was obviously to disarm the experience of others’ degradation by using humour, self-irony, and by catching up with others. By making responses to others in such ways, the participants took the sting out of any possible degradation, so that the effect more or less “peeled off”. Another 58-year-old woman suffering from pain in her knees and ankles, reflected in the interview upon sometimes being teased kindly with laughter when making excuses to her colleges about her limitations in her practical work as a shop assistant. However, she caught up with them and teased them back:

Maybe the people at work see that I struggle with some things that they manage easier. For example, walking down a stone staircase carrying cardboard (packaging) and that kind of thing. I always say ‘I will just put it (the packaging) here’ when I am unpacking goods. I don’t take one box down the stairs at a time. The exercise is good for me, but I don’t do it because of my hurting knee and the ankles. They laugh then. Oh well. But then I tease them afterwards, ‘I see that you don’t do it either’. So, I get it.

Comparing themselves with others brought forth a recognition among participants that they were not so different from others after all. Reciprocal teasing contributed to a feeling of “being in the same boat”. These kinds of experiences made the participants feel that they could relate to others on equal footing.

In addition, disarming the experience of others’ degradation was also about attempting to influence others’ perceptions that had the potential to shape the participants’ self-understanding, such as distracting attention. Most of the participants related that they were attempting to downplay the body parts they wanted to hide. However, some were very conscious about distracting the attention of others by highlighting a part of the body they were satisfied with, such as their face. A 46-year-old woman expressed her strategy like this:

I love to decorate myself! I probably have 50 different pairs of earrings. I change them every day. I never wear the same pair two days in a row … It is probably to do with taking the focus away from my body. They catch your eye … they sparkle a bit, don’t they? So then, people look at my face instead of looking at my body.

To decorate themselves, for example with conspicuous earrings was a strategy that enabled the size of their body to move into the background. These small diversionary manoeuvres made the participants feel that they had a degree of control when encountering others, and in this way, they seemed not totally left to devaluation by others. Moreover, they might function to restore the perceived relational unbalance between themselves and the world.

#### Opposing the internalization of degradation

Another strategy that appeared in the interviews was that the participants seemed to defend themselves by oppose internalization of others’ degradation. Among the participants, degradation had the potential to affect their self-understanding negatively, but if not internalized, it would not be “effectuated”.

The strategy of participants seemed to be to keep degradation at a distance by letting it bounce off immediately. By challenging others’ simplified understanding of what was needed to lose weight, the participants were able to recognize their own and others’ efforts to overcome obesity. During the interview a 58-year-old woman established a clear distance from the cultural prejudice that people with obesity have little will-power:

It seems that most people think it is just a matter of deciding to eat less and exercise more, and then the problem will be fixed. But then it isn’t as easy as that. One is perhaps seen as undisciplined, though being on a chronic diet requires a fair amount of discipline. And it takes at least two years to stabilise the weight. And while you are in that stabilising phase, it is pretty tough. It actually demands a lot of will-power to endure that, which I don’t think everybody understands.

Western cultures judge those living with obesity as having little will-power. For the participants it was important to support themselves, and stand up against such a judgemental attitude. By keeping the devaluation at a distance, the participants seemed to avoid self-devaluation. Further, self-support meant that the participants felt pride in their inner qualities.

### Reorienting the pivot in life

The participants related that their body and weight, and possibilities for weight loss had occupied their attention year after year. This had caused a feeling of stagnation in life and inevitable experience of loss associated with not achieving their goals or dreams. However, participants also expressed being in a process of reorienting their pivot in life, focusing on living well with their condition.

#### Rethinking how life can be lived

Participants reflected on the possibilities for living a good life, given that they had experiences of loss in life related to their large body. Despite these experiences, they reflected that they could still have a good life, if they could only adjust their dreams. A 56-year-old man realized during the interview that he had to reorient his desire about climbing mountains to another activity that would fit his current body function better.

I will always be very big, but as long as my body can take me up some mountaintop … (stops in the middle of the sentence, thinks for a moment before he goes on), or at least down to the sea and along the beach, I will be very satisfied and happy.

Dwelling on what the body could actually contribute, turned the participants’ lives towards satisfaction and a form of happiness.

Rethinking how life could be lived also seemed to be based on reflections of what life actually should be in the future. They questioned whether their future life should concentrate only on waiting for weight loss. They had started to realize that life now was so much more than that. Participants experienced for example feelings that a long-awaited life had come true, despite having the same bodily limitations as earlier. A young woman, 24 years old, previously could not believe that she would ever have a boyfriend with such a body as she had, but put this in connection with accepting herself and her body.

I think I am on the right track. I have a boyfriend. I never thought I would have one. And I am starting to accept my body as it is, both the good and the bad … . In a way, I have accepted that this is the size I am right now, and that is who I am.

At the same time, there was recognition that their large body was good, but also had challenging aspects. Nevertheless, participants came to understand what mattered in life. Realizing that life could really be *lived*, while having a large body was an understanding they acquired, as they reflected upon their future possibilities.

#### Rethinking what is important in life

Reorienting in life also meant changing beliefs of what was important in life. The participants seemed to be in a process where they eventually let go of old ideas about what was important in life, and yet took their life situation to a new assessment. At the time of the interviews, some participants expressed that they had gradually understood that life could be good, even while living with a large body. However, moving from knowing what was good for them to believing in it had been so difficult, that it required them to gain another understanding of what was important. A 24-year old woman’s reflections revealed that she now considered *her process* towards long-term lifestyle changes to be more important than simply aiming for a slim body: “*I see that my weight is too high, but when diet and exercise is all in place, I will have a well-proportioned body, which I see as healthy*.”

The participants seemed to have realized that they had to decide for themselves what their life and mind should be filled with. Being constantly occupied with slimming for so many years had drawn the focus away from feeling good about themselves. Some valued gradually feeling good about themselves as being more important than having a slim body but coming to that conclusion had been a mental process lasting years. A 55-year-old woman gave the impression that she felt she had lost many years of her life because of constantly feeling sad about herself, while being occupied with losing weight:

I can remember that when I weighed 60 or 65 kg. I wasn’t happy then. When I weighed 70 and 75 kg, I wasn’t happy either. And when I was 80 and 85 kg, I wanted to lose weight too. If I was 85 kg now, I would be happy, is what I say now. … I have decided now that I am not going to have as much focus on dieting. Well, focus, but on myself instead … on feeling good.

The participants expressed their experience of having suffered for so many years to no avail. In retrospect, they seemed to regret that they had let themselves believe that slimness and happiness were so closely connected.

## Discussion

The aim of this study was to gain deeper insight into how people struggling with obesity handle their life situation by addressing how well-being unfolds. We found that handling life with obesity could be seen as an evolving process while experiencing aspects of well-being, including: *coming to terms with the body, restoring the broken relational balance* and *reorienting the pivot in life*. These three thematic findings are now interpreted and discussed in light of earlier research, but mainly in the light of the conceptual framework of Galvin and Todres (2013) about well-being. From this theoretical perspective, the three thematic findings are abstracted into a main theme. We consider the movement towards well-being as a struggle towards an experience of balance to make bearable living. Striving to make living bearable is an expression of how people with obesity struggle to find their own balance and harmony in existence, which is intertwined with one’s life situation (Gadamer, 1996). This discussion has been arranged according to the abstracted main theme.

### Striving to make living bearable

As we have presented, the abstracted findings from our study show that the participants seemed to struggle towards an experience of balance to make living bearable, within their perceived limitations. It appears that the participants’ reflections upon their bodies, relations and selves fluctuated between opposite poles. There also seems to have been movement between not living one’s life to the fullest and expanding one’s living space. In light of the conceptual framework of Galvin and Todres (2013), this can be seen as moving between illness and health. According to Merleau-Ponty (2002), bodily being involves a certain ambiguity. The ambiguity regarding one’s body, relations and self is described in several phenomenological lifeworld studies within the obesity field (Groven et al., 2015, 2017; Westland Barber, 2017). Our research has revealed that aspects of well-being can be found within the struggle of people with obesity to make living bearable. Moreover, our study adds to the literature on how this positive possibility becomes a resource for life when living with obesity. Thus, human beings cannot be seen as finished, but always in a process (Galvin & Todres, 2013) Ambiguity is an essential part of being human (Galvin & Todres, 2013). Therefore, the movement between opposite poles can be understood as a way of being in the world, as the participants seem to strive for the experience of a greater level of well-being in their life.

As shown, the participants in our study seem to be in a process of coming to terms with their bodies, through discovering their body as a prerequisite for life and by negotiating with the body’s possibilities. The concepts “discovering the body” and “negotiating with the body” reveal the participants’ realization that life itself can only be experienced through the body, despite experiencing their body as an opponent to living. According to previous research, struggling with obesity can lead to a feeling of not having full access to life, so life can be experienced as being “put on hold” (Haga et al., 2019). However, in the present study, we discovered that the participants found themselves negotiating ongoing tension between restriction and freedom of action because of their perceived bodily limitations. As we see it, this process seems to involve a gradual movement towards adjusting to the reality of their bodies. They gradually learned to live with this tension by acknowledging their bodily experiences as a source of insight and meaning in their life. Further, the findings show an aspect of well-being arising from acknowledgement of their body. The reflections contribute to the participants’ consciousness about how they should adjust their life to make living bearable. Our findings are related to a study about making sense of long-term bodily changes following bariatric surgery. In that study, Groven et al. (2015) described the struggle as an inevitable balancing process. According to the terminology of Galvin and Todres (2013), the movement between limitations and possibilities when coming to terms with the body can be referred to as an experience of “abiding expanse”. This concept describes an aspect of well-being as stretching between a sense of at-homeness, and a sense of being invited into an exploration of something new. We suggest that the experience of well-being becomes a foundation that enables the person to move further. Hence, the reflections based on the experience of coming home, i.e., being deeply rooted in oneself and one’s bodily vulnerabilities, appear as intertwined with the possibilities for life and health. As such, well-being seems to be strengthened by an individual’s ability to reflect on their bodily vulnerabilities, which again appear as a foundation for exceeding one’s limitations. It seems particularly important that the foundation on which people with obesity stand is to *be in* their body, and to acknowledge what it means to take both possibilities and limitations into account. Furthermore, the important things in life arise from this basis.

This study has revealed participants’ reflections and strategies on how they deal with the feeling of degradation. As we see it, their experience of a judging gaze from both individuals and society, reveal a sense of a broken relationship. The participants in our study seem to strive for balance in relation to others by using certain strategies. Moreover, they seem to move against a deeper experience of well-being as they manage to defend themselves from the underlying judgemental tone of others.

As previous studies have shown, people with obesity strive to fit in, and yet feel a second-rate citizen and human being, as they do not comply with prevailing social norms in the culture of adequate body weight and shape (Tomiyama, Carr, Granberg, Major, Robinson, Sutin & Brewis, 2018). Forms of intersubjectivity can humanize or dehumanize the individual and have a positive or negative impact on well-being (Hemingway, 2011). Standing outside the accepted fellowship can lead to a sense that one’s existence is not wanted (Galvin & Todres, 2013). However, in our study we see that within the feeling of strangeness, the participants aim to disarm or draw the attention away from what is different, or what they want to hide by how they communicate (both said and unsaid). Instead, it seems that they attempt to draw attention towards what unites them with others. Thus, the experiences of familiarity and partnership. contribute to the constitution of a symmetrical relationship. Therefore, an aspect of well-being seems to lie in the participant’s reflections between difference and sameness, strangeness and familiarity, which leads attention towards what they have in common with others, and not the opposite (Galvin & Todres, 2013).

Another way of restoring the broken balance in relation to others seems to be when the participants oppose internalization of others’ degradation. The participants seem to struggle with the sense of self as being a failure because of the cultural prejudice that people with obesity have little will-power. Other researchers have confirmed that people with obesity seem to develop an identity in the experience of what others mean about them (Malterud & Ulriksen, 2010). However, we can see that the participants in our study seemed to take a meta-perspective. According to Fuchs (2002) this perspective allows a kind of self-distancing, which is crucial to cope with self-devaluation. Therefore, the participants’ ability to take a meta-perspective when encountering society’s condemnation seems to strengthen their opposition. Moreover, we found traces of pride associated with the person’s inner qualities within their reflections. This finding agrees with other studies in the obesity field (Salemonsen, Hansen, Førland, & Holm, 2018; Ueland, 2019). The importance of highlighting these traces as building blocks to liberate oneself from shame is reported to be essential to cope with cultural influences (Ueland, 2019). As we see it, the self-reflective attitude described in the present study might be an aspect of well-being, as reflection as such provides an awareness of oneself in relation to the phenomenon for consideration (Hörberg, Galvin, Ekebergh, & Ozolins, 2019) The aspect of well-being lies in the breakthrough of realizing “I am all this and more” (Galvin & Todres, 2013). Thus, our findings indicate that the reflection process gradually leads to a change in the participant’s self-understanding (Hörberg et al., 2019). Earlier research confirmed that the processes related to developing embodied knowledge is deeply personal and often entail an existential experience of oneself (Natvik, Råheim, Andersen, & Moltu, 2018; Toft & Uhrenfeldt, 2015). What our study offers is that when self-devaluation does not seem to be at stake any longer, a sense of freedom emerges, and creates an interpersonal space for developing an identity rooted in oneself. As we see it, the experience of well-being provides the participants the freedom to build a new understanding of themselves that enables them to keep their dignity in the face of others. Consequently, a transformed relationship to oneself, based on an experience of well-being, seems to be important to people with obesity, to make living bearable in a judgemental society.

Our study strongly emphasizes the participants’ considerations regarding a reorienting in their pivot in life. The reflections made by several of the participants in our study seem to reveal that they are in a process of giving their current life and future a new pivot. Therefore, this reorientation in life includes rethinking how their life can be lived in the future. As some of the participants reflected on possibilities for living a good life—within their bodily limitations—at the same time they seemed to adjust their ambitions to their current level of bodily capability to make living bearable. Research on living with a chronical illness confirms that opting for less favourable choices sometimes also means achieving a balance in life (Johansson et al., 2015). Groven et al. (2017) referred to the concept of “flow”, which is described as a state in which the lived body feels in tune with the environment, as possibly providing new hope for the future. Galvin and Todres (2013) linked the movement towards the future to being “at one” with the present moment by constitution of a sense of meaningful purpose. As we can see, in our study, meaningful purpose was constituted at the moment the participants experienced a sense of completeness and satisfaction.

In some sense, the adjustment of future ambitions represents a temporal stagnation and a lack of temporal mobility that in one way affects one’s understanding of having access to a future with possibilities. However, according to aspects of well-being there seems to be a dialectic between accepting what is lost and orienting towards a meaningful future. This dialectic can be described with the concept *renewal* (Galvin & Todres, 2013). As we have seen in the present study, the feeling of “the future is now” appeared as one of the participants described accepting herself and her body in connection with unexpectedly reaching one of her life goals, without losing weight. The experience of “the future is now” might be seen as renewal, as this well-being experience unifies future orientation and present centeredness. Accepting oneself and one’s body seem to give a kind of “aliveness” to a sense of the present and provide a strong base for movement towards the future (Galvin & Todres, 2013).

Furthermore, our findings show that the participants in our study seemed to be in a process of rethinking what was meaningful in their life. They seemed to consider a shift in their life’s pivot. They had believed for many years that slimness meant a happier life. Research has shown that if only a fixed ideal of the body is valued, it might appear difficult to be happy outside the norm (Bahra, 2018). Being constantly occupied with slimming led to an experience of having lost many years of life, to no avail. They now concentrated more on living in the present and feeling good about themselves. Self-compassion seems to be pivotal in relation to well-being when confronted with challenges in life (Gustin, 2017). Hence, the literature confirms that the lifeworld perspective highlights that life is not an outcome, it is a process that is deeply personal and points to the existential domain (Natvik et al., 2018). A change in perspective is referred to as a change in attitude towards life, which involves a decision of the self (Natvik et al., 2018). According to Galvin and Todres (2013) the decision of the self seems to point towards an aspect of well-being. The ability to make decisions indicates that the person with obesity holds a certain power and capacity, which might provide life-heading and life-forcing qualities of being an active agent in the world (Galvin & Todres, 2013). As we see it, the well-being experience might enable the person with obesity to concentrate his/her energy more towards living well with their condition.

### Methodological considerations

In lifeworld studies, openness and sensitivity is imperative (Dahlberg et al., 2008). We have described how we have approached the phenomenon as it presented itself, instead of imposing preconceived ideas or hypotheses. Three themes were developed as offering interesting perspectives that, in combination, could shed light on the existential experiences of people living with obesity. Moreover, given that we are a mixed group of researchers, the analysis process included ongoing discussions of alternative interpretations before reaching a consensus. This was done to enhance the trustworthiness of our findings. However, meanings are never finally complete, but ambiguous and tentative, leaving the analysis open for other interpretations (Dahlberg et al., 2008).

We have presented the findings together with the participant’s quotations, a description of the analytic process, and clarified an example of a structural analysis (Table II). This gives the reader the possibility to follow a researcher’s reasoning throughout the whole study and should contribute to strengthen the study’s validity (Dahlberg et al., 2008). However, validity in phenomenological studies may also be verified by the writer’s ability to create reflections that touch, guide and stir the reader to the extent that one is able to evoke the reader’s recognition (Van Manen, 2014).

The diversity of the sample of 21 participants representing different ages, both men and women of different backgrounds offered rich and nuanced descriptions of the phenomenon of interest. We consider it as a certain strength that both genders are well represented, and that half of the sample were employed (vs. unemployed). The more variations and nuances in the data, the more likely it is to see clear patterns of meanings in the phenomenon (van Wijngaarden, Meide, & Dahlberg, 2017). Together with the descriptions of the existential experiences and the background information, we suggest that the provided meaning structure of the phenomenon gives insight into life-as-it-is-lived, and allow transferability to be discerned, by considering the culture and the context.

The participants were recruited when entering a health-promoting program and that might be a possible limitation. Their utterances expressing aspects of well-being might have been influenced by a “flow” of positivism and strength, as they were filled with hope and expectations and felt that they were standing on the threshold to a new life. However, this program was not a subject for the interviews. Moreover, the participants were recruited in the most appropriate way to find those whose experiences could provide insight into the phenomenon of struggling with obesity, given the sensitive topic and considerations made after other researcher’s difficulties in more general recruiting (Westland Barber, 2017). We are nevertheless aware that another sample strategy could have provided other findings.

## Conclusions

This study has gained insight into how people struggling with obesity strive to experience well-being. Well-being in our findings is described as a struggle to achieve a bearable living. This struggle is expressed through the study by how people strive towards coming to terms with their bodies, towards restoring the broken relational balance and towards reorienting their pivot in life, focusing on living well with their condition. These movements within the experience of well-being can be understood as a dialectic between vulnerability and freedom.

Freedom emerged as a possibility to orient towards a better life, where the weight is not allowed to become burdensome. However, this freedom takes place in a vulnerable space, where the person living with obesity experience an internal and external devaluing pressure. The dialectic between experiencing freedom and experiencing vulnerability coincide, and points towards “dwelling-mobility”, as a way of being-in-the-world. We suggest that well-being as a dialectic between vulnerability and freedom might become a health-facilitating experience for people struggling with obesity.

### Implications for practice

We suggest a change in focus towards health and well-being when approaching the challenge of people struggling with obesity. As mentioned, the standard biomedical approach to obesity primarily involves an illness perspective. In particular, our findings point towards the need to understand the individual efforts of people with obesity to regain balance and well-being in their lives, including the dialectic between vulnerability and freedom. Health professionals have possibilities to support their patients’ health-facilitating processes. However, they need to be aware of their patient’s expressions of well-being if they can support them. Therefore, we suggest that people with obesity involved in treatment processes should be invited into a dialogue, with the purpose of reflecting on how they handle their challenges and make sense of their ongoing bodily challenges in everyday life. We believe that reflections on possibilities and obstacles in a patient’s lifeworld can give rise to a deeper understanding of what really matters for the person with obesity. Further research should focus on what health professionals experience when encountering people with obesity, and how they understand the existential struggle of living and handling life with obesity.
